# Biodegradable FeMnSi Sputter-Coated Macroporous Polypropylene Membranes for the Sustained Release of Drugs

**DOI:** 10.3390/nano7070155

**Published:** 2017-06-24

**Authors:** Jordina Fornell, Jorge Soriano, Miguel Guerrero, Juan de Dios Sirvent, Marta Ferran-Marqués, Elena Ibáñez, Leonardo Barrios, Maria Dolors Baró, Santiago Suriñach, Carme Nogués, Jordi Sort, Eva Pellicer

**Affiliations:** 1 Departament de Física, Universitat Autònoma de Barcelona, E-08193 Bellaterra, Cerdanyola del Vallès, Spain; jordina.fornell@uab.cat (J.F.); juandesirvent@gmail.com (J.d.D.S); marta.ferranmarques@gmail.com (M.F.-M.); dolors.baro@uab.es (M.D.B.); Santiago.Surinyach@uab.cat (S.S.); eva.pellicer@uab.cat (E.P.); 2 Departament de Biologia Cel·lular, Fisiologia i Immunologia, Universitat Autònoma de Barcelona, E-08193 Bellaterra, Cerdanyola del Vallès, Spain; jorge.soriano@uab.cat (J.S.); elena.ibanez@uab.cat (E.I.); lleonard.barrios@uab.cat (L.B.); 3 Institució Catalana de Recerca i Estudis Avançats (ICREA), Pg. Lluís Companys 23, E-08010 Barcelona, Spain

**Keywords:** biodegradable material, porous membrane, drug delivery, hybrid material

## Abstract

Pure Fe and FeMnSi thin films were sputtered on macroporous polypropylene (PP) membranes with the aim to obtain biocompatible, biodegradable and, eventually, magnetically-steerable platforms. Room-temperature ferromagnetic response was observed in both Fe- and FeMnSi-coated membranes. Good cell viability was observed in both cases by means of cytotoxicity studies, though the FeMnSi-coated membranes showed higher biodegradability than the Fe-coated ones. Various strategies to functionalize the porous platforms with transferrin-Alexa Fluor 488 (Tf-AF488) molecules were tested to determine an optimal balance between the functionalization yield and the cargo release. The distribution of Tf-AF488 within the FeMnSi-coated PP membranes, as well as its release and uptake by cells, was studied by confocal laser scanning microscopy. A homogeneous distribution of the drug within the membrane skeleton and its sustained release was achieved after three consecutive impregnations followed by the addition of a layer made of gelatin and maltodextrin, which prevented exceedingly fast release. The here-prepared organic-inorganic macroporous membranes could find applications as fixed or magnetically-steerable drug delivery platforms.

## 1. Introduction

The most widely used forms of drug delivery are oral ingested tablets, injection, transdermal patches, and implantable fixtures. Once administered, controlling the rate at which the drug is released in the body and its transport to the desired location becomes crucial. One very important requirement is that the drug must display some preferential selectivity to the target tissue or organ in order to avoid side effects due to systemic exposure of healthy organ systems to the drug. This is of utmost importance in cancer treatment and has led to the discovery of many innovative approaches for drug delivery, frequently taking advantage of concepts from nanotechnology [1,2,3].

Polymeric materials are particularly amenable for medical applications because they offer an interesting combination of properties, such as diffusivity, permeability, biocompatibility, and solubility. In addition, they can respond to pH or temperature changes. The diffusion, dissolution, permeation, and swelling characteristics of polymeric materials have been exploited to obtain a constant release of entrapped molecules [4]. Pore-containing polymer systems have emerged as a new class of efficient drug delivery platforms [5,6,7]. By controlling the size and shape of the material, the number of “reservoirs” and their volume, the wall thickness, and the surface chemistry, permeability, and resorption rate, porous polymers enable better control of the vectorisation and the release kinetics of the drug. 

Traditional, degradation-resistant polymers for implantable medical devices, such as polytetrafluoroethylene (PTFE), polyether ether ketone (PEEK), poly(methyl methacrylate) (PMMA), polyethylene, and silicones have been frequently associated with clinical problems, including microbial biofilm formation and medical device related infection, encrustation, poor biocompatibility, and low lubricity [8,9,10]. For this reason, bioresorbable polymer implants, employed in tissue engineering, absorbable sutures, arterial stent coatings and implants for controlled drug delivery have been devised. 

For the therapeutic delivery of molecules, the polymer can act as a passive entity that hosts the drug and ensures its sustained release (e.g., subcutaneous patch) or can be envisioned as a micro-robotic platform that enables both transport of the molecule to the target site and its subsequent release. Guidance of micro-robotic platforms under the action of magnetic fields is a promising approach because they are capable of penetrating most materials with minimal interaction, and are almost harmless to human beings even at relatively high field strengths (provided no high-frequency AC magnetic fields are used). Magnetic fields have been successfully used to wirelessly manipulate microdevices of various sizes and shapes [11,12]. Polymers have been already combined with magnetic materials such as magnetic nanoparticles (MNPs) to obtain carriers consisting of an inorganic core, composed by the MNP, and a biocompatible polymer coating, in which functional ligands (e.g., targeting agents, therapeutic agents, permeation enhancers) can be integrated. Magnetite (Fe_3_O_4_) and maghemite (γ-Fe_2_O_3_) MNPs have been mostly used as cores. Magnetic layer-by-layer (LBL) capsules consisting of multilayer polymeric capsules containing MNPs are another example of polymer-magnetic material hybrids [13]. 

Here, we propose the combination of a porous polymeric backbone (polypropylene, PP) and a sputtered ferromagnetic Fe-based metallic layer for biomedical applications. There are a few examples of polymeric membranes sputtered with single metals like Pt and Ni for different purposes [14,15]. In this work, a ternary alloy (FeMnSi) is sputtered onto the biodegradable, macroporous PP membrane [16] for the first time. Among ferromagnetic metallic materials, Fe-based ones are very convenient because of their cytocompatibility and magnetic response. During the last few years, Fe has been typically co-alloyed with other elements, such as Mn, Pd, Si, C, or P, to increase the degradation rate of Fe-structures without compromising their mechanical integrity and their cyto- and hemocompatibility [17,18,19,20]. It is, therefore, the aim of this work to produce porous, fully-biodegradable, magnetic organic-inorganic polymer-metal hybrids that could be used either as implantable patches or as steerable micro-robotic platforms if appropriately miniaturized. With this purpose, commercial macroporous PP membranes (pore size ~450 nm) were coated with FeMnSi to confer magnetic properties to the membranes whilst avoiding clogging of the pores. The microstructure, morphology, biodegradability, and cytocompatibility of the FeMnSi-coated membranes were studied and compared with those of Fe-coated membranes (taken as a reference). These magnetic organic-inorganic hybrids were loaded with Transferrin-Alexa Fluor 488 (Tf-AF488) to investigate its distribution within the porous skeleton, its kinetics of release, and its cellular uptake.

## 2. Materials and Methods

### 2.1. Preparation and Characterization of the Macroporous Fe- and FeMnSi-Coated PP Membranes

#### 2.1.1. Sputtering of Fe and FeMnSi on Macroporous PP Membranes

Pure Fe and FeMnSi thin films were grown by magnetron sputtering using a ATC Orion 5 from AJA International equipment (N. Scituate, MA, USA). The films were sputtered on PP membranes of 47 mm in diameter and a pore size ~450 nm (purchased from Sigma-Aldrich, St. Louis, MO, USA). The sputtering process to coat the PP membranes with Fe was carried out at 100 W in DC using a Fe target (99.9% purity) for 15 min. To coat the PP membranes with a targeted composition of Fe-14Mn-4Si (wt %), simultaneous sputtering from a Fe target at 200 W in RF and a Fe-30Mn-6Si-1Pd (99.95% purity) target at 100 W in DC was carried out for 5 min. To determine the thickness of the resulting coatings, Fe and FeMnSi thin films were sputtered on flat Si substrates under the same conditions as for the membranes. The thickness was assessed with a 3D Optical Surface Metrology System (DCM 3D) from Leica which combines confocal and interferometry technology. The deposition time was adjusted to obtain films with similar thickness and magnetization response.

#### 2.1.2. Structural and Magnetic Characterization of the Fe- and FeMnSi-coated PP Membranes

The morphology and chemical composition of the materials were investigated by field emission scanning electron microscopy (FE-SEM, Zeiss, Oberkochen, Germany) using a Merlin Zeiss microscope equipped with an energy dispersive X-ray (EDX) detector. Transmission Electron Microscopy (TEM) characterization was carried out on a JEOL JEM-2011 (Tokyo, Japan) microscope operated at 200 kV. For TEM observations, cross-sections were prepared by embedding the FeMnSi-coated PP membrane in an epoxy resin followed by cutting thin slices with an ultramicrotome (Leica EM UC6, Leica Microsystems Ltd., Milton Keynes, UK) using a 35° diamond knife at room temperature (RT).

X-ray diffraction (XRD) patterns were recorded on a Philips X’Pert diffractometer using Cu Kα radiation, in the 40°–60° 2θ range. For XRD measurements, the targeted compositions were sputtered following the same conditions on flat Si/Ti/Cu substrates for 15 min to increase the amount of material on the diffraction focal plane. Hysteresis loops were acquired at RT using a vibrating sample magnetometer (VSM) from Micro-Sense (from LOT-Quantum Design, Darmstadt, Germany), varying the magnetic field from −20 to 20 kOe.

#### 2.1.3. Biodegradability Studies

In order to evaluate the release of metal cations from the samples, coated PP membranes with an exposed geometrical area of 0.5 cm^2^ were introduced in sterilized plastic containers filled with Hank’s balanced salt solution (HBSS, Sigma-Aldrich, Nuaillé, France). A blank PP membrane (without coating) was also immersed in HBSS for comparison purposes. The containers were then sealed and placed in a thermostatic bath at 37 °C. After 15 days the solution was pipetted off and placed in tube tests for inductively coupled plasma-mass spectroscopy (ICP-MS) measurements, which were carried in an Agilent 7500 ce apparatus (Midland, ON, Canada). The ratio between the HBSS volume to geometrical sample surface was 25 mL cm^−2^, in accordance with the ASTM-G31-72 standard [21]. Notice that this is an upper threshold since the porosity (i.e., the real surface area in contact with the solution) is not taken into account.

#### 2.1.4. Cell Culture

Cellular studies were conducted using a tumoral human mammary epithelial cell line, SKBR-3 American Type Culture Collection (ATCC, Manassas, VA, USA). The cells were cultured in McCoy’s 5A modified medium (Gibco, Paisley, UK) supplemented with 10% fetal bovine serum (FBS, Gibco, Paisley, UK). The cell line was maintained at 37 °C and 5% CO_2_ (standard conditions). The culture medium was refreshed every 72 h.

#### 2.1.5. MTT Assay

To evaluate a possible cytotoxic effect of the Fe- and FeMnSi-coated PP membranes, cell viability (metabolic activity) was tested by MTT (3-(4,5-dimethylthiazol-2-yl)-2,5-diphenyltetrazolium bromide) assay (Sigma-Aldrich, Nuaillé, France).

A total of 10,000 cells were seeded in each well of a 24-well dish in order to carry out the experiment. At 24 h after seeding, pieces of 3 mm × 3 mm (9 mm^2^) were cut from the Fe and FeMnSi-coated PP membranes, cleaned with ethanol, and introduced individually in Millicell^®^ 8 µm cell culture plate inserts (Millipore, Bedford, MA, USA), which were then placed inside the wells of the 24-well dish. This approach was used to prevent the membranes to crush the cells. After one week, the MTT assay was performed and the absorbance was recorded at 540 nm using a Victor 3 Multilabel Plate Reader (PerkinElmer, Waltham, MA, USA). For each treatment, viability was compared with that of cells incubated without the membranes (control). Three independent experiments were performed for each condition. 

### 2.2. Functionalization of the Macroporous FeMnSi-Coated PP Membranes

In order to functionalize the FeMnSi porous membranes, 9 mm^2^ cuts were loaded with Transferrin-Alexa Fluor 488 (Tf-AF488, 1 mg mL^−1^, Molecular Probes, Eugene, OR, USA), maltodextrin (25 mg L^−1^) and gelatin from bovine skin (0.25 mg mL^−1^) purchased from Sigma-Aldrich (Nuaillé, France). Transferrin was used in order to target SKBR-3 cells as the transferrin receptor has shown to be overexpressed in SKBR-3 cells [22]. The maltodextrin and gelatin were used as co-gelling agents. Fifteen µL of the previous solution was added dropwise onto the surface of the coated membranes. The wetted FeMnSi-PP porous membranes were placed in a vacuum desiccator (0.5 mbar) for 30 min at RT to force the gel to penetrate into the network. This process was done once or repeated three consecutive times. After each impregnation, the membranes were rinsed with HBSS. In some cases, a drop of maltodextrin and gelatin was placed on top of the loaded membranes to partially block and hence delay the release of Tf-AF488. Table 1 lists the steps followed for each sample. Sample 1 was loaded with Tf-AF488 once and dried in vacuum. Sample 2 was also loaded once, rinsed in HBSS, and dried in vacuum. Subsequently, a stopper made of gelatine and maltodextrin was added on top of the membrane to prevent a fast release. Sample 3 was loaded three times with Tf-AF488, washed with HBSS after the last load, dried in vacuum and blocked twice with the stopper.

### 2.3. Characterization and Performance of the Tf-AF488-Loaded Membranes

#### 2.3.1. Distribution of Tf-AF488

To evaluate the distribution of the Tf-AF488 inside the membranes, the photoluminescence response of the materials was studied by confocal scanning laser microscopy (CSLM). The samples were mounted on Ibidi culture dishes (Ibidi GmbH, Martinsried, Germany). For 3D reconstruction, the samples were visualized with a TCS-SP5 CSLM microscope (Leica Microsystems CMS GmbH, Mannheim, Germany) using a Plan Apochromat (Leica, Mannheim, Germany) 20.0×/0.70 (dry) objective. The membranes were excited with a green diode laser (488 nm) and the fluorescence of the Tf-AF488 was detected in the 500–560 nm range. The PP membranes (non-fluorescent) were imaged in the reflected light mode from an argon laser (488 nm) and subsequently detected in the 480–495 nm range. Stacks were collected at every 0.5 µm along the material’s thickness. The three-dimensional images were processed by using Surpass Module in Imaris ×64 v. 6.4.0 software (Bitplane; Zurich, Switzerland). A set of 15 regions of interest (ROIs) of 400 μm^2^ each was used to analyze the mean fluorescence intensity of the samples in relation to the emission wavelength. 

#### 2.3.2. Release of Tf-AF488

The kinetics of Tf-AF488 release from the loaded membranes was studied by monitoring the fluorescence signal of Tf-AF488 using a fluorimeter (Varian Cary Eclipse, Palo Alto, CA, USA). To carry out the fluorescence measurements, 1 mL of HBSS and one single Tf-AF488-loaded membrane piece (9 mm^2^) were placed inside a cuvette. A stirring bar was added to homogenize the solution. Samples were excited at 488 nm and emission was measured every 5 min at 517 nm, as this was the point of maximum emission of Tf-AF488. Two replicates for each condition were prepared to evaluate the reproducibility of the absorption and release of Tf-AF488. After fluorometric measurements, the membranes were imaged by confocal microscopy to confirm the total release of Tf-AF488. 

#### 2.3.3. Tf-AF488 Uptake by Cells

A total of 50,000 cells were seeded in each well of four-well dishes. After 24 h, the membranes previously charged with Tf-AF488 were added in a Millicell^®^ 8 µm cell culture plate insert and images of the cells were taken at different times of incubation using a fluorescence microscope (Olympus IX70, Olympus, Hamburg, Germany).

## 3. Results and Discussion

### 3.1. Characterization of the Fe- and FeMnSi-Coated Membranes

#### 3.1.1. Structural and Morphological Characterization

**Figure 1:**
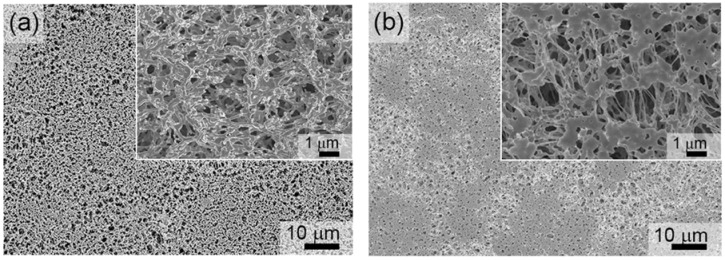
SEM image of PP membranes coated with (**a**) Fe and (**b**) Fe-14Mn-4Si alloy. The insets are higher magnification images.

To verify that the porosity of the resulting platforms was not compromised by the sputtering process, SEM imaging was carried out on the Fe- and FeMnSi-coated membranes. Pores were clearly visible after Fe sputtering on the PP membrane at 100 W for 15 min (see Figure 1a). A representative SEM image of the PP membrane co-sputtered from Fe and FeMnSiPd targets for 5 min is presented in Figure 1b. In this case, the co-sputtering process (i.e., applied voltage), was adjusted to render a nominal composition of Fe-14Mn-4Si (in wt %). Additionally, the deposition time was adjusted to obtain nanocoatings of similar thickness (~70 nm). Although the co-sputtering resulted in a decrease of the porosity degree (i.e., slightly smaller pores and thicker pore walls), the coated membrane is still rather porous (see the inset of Figure 1b). It has been reported that the addition of Mn within the solubility limit of Fe reduces the standard electrode potential of Fe, thereby making it more susceptible to corrosion [23,24,25]. The composition of the co-sputtered film coating the PP membrane measured by EDX.

The influence of the alloying elements (Mn and Si) on the lattice structure of the resulting nanocoatings was investigated by X-ray diffraction (Figure S1). Note that Fe and FeMnSi were sputtered onto a non-porous Cu-coated Si substrate instead of the porous PP membrane with the aim to increase the amount of material on the diffraction focal plane. For the FeMnSi layer, the slight shift of the (110) diffraction peak characteristic of the body-centered cubic (bcc) phase of Fe towards lower diffraction angles is indicative of the substitutional solid solution. No additional peaks belonging to Mn, Si, or oxides could be observed, corroborating the formation of a single-phase nanocoating.

**Figure 2:**
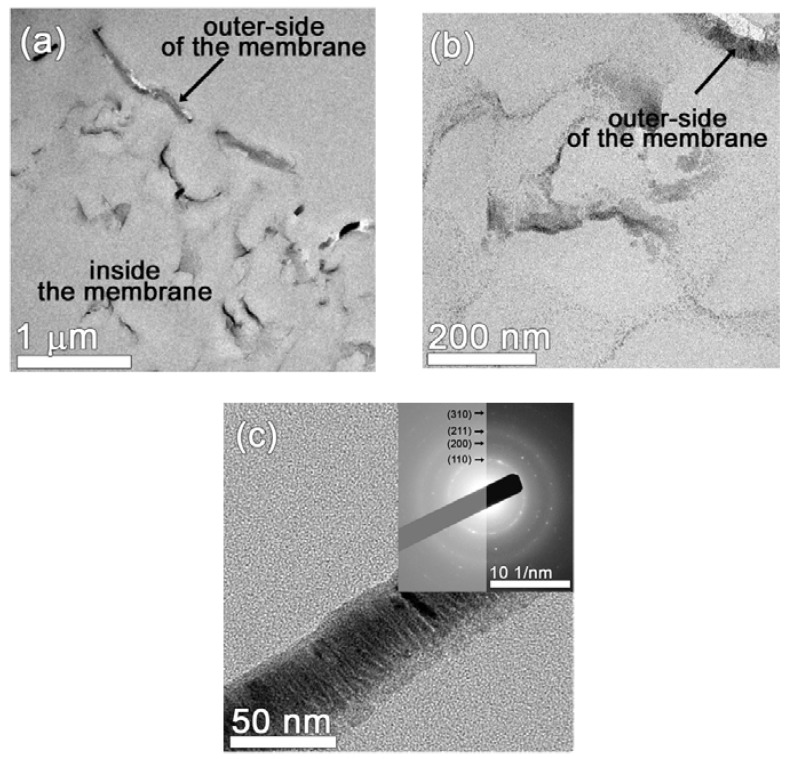
Cross-section TEM image of the FeMnSi-coated PP membrane (**a**) showing the outer and inner FeMnSi coating (**b**) higher magnification image taken inside the membrane; and (**c**) the FeMnSi layer grown on top of the membrane of ~40 nm in thickness. The inset belongs to the corresponding SAED pattern.

To gain further information about the structure and morphology of the coated membranes, TEM imaging was carried out on the FeMnSi-coated membrane. In the low magnification TEM image presented in Figure 2a, a discontinuous Fe-based layer can be observed at the outer side of the PP membrane. Furthermore, it can also be observed that the FeMnSi penetrates inside the PP membrane up to ~2 μm. A higher magnification image (Figure 2b) reveals that, besides the thicker FeMnSi layer grown on top of the membrane, nanometer-sized crystals surrounding the pores can be observed within the first few hundred nanometers from the surface. From Figure 2c an average thickness of 40 nm of the top layer can be measured. The selected area electron diffraction (SAED) image (inset of Figure 2c) confirms the crystalline nature of the outer Fe-based layer. The interplanar distances’ values are slightly larger than the theoretical bcc-Fe interplanar distances due to the presence of Mn atoms dissolved in the Fe lattice. The spots/rings of the SAED pattern belong to (110), (200), (211), and (310) planes.

#### 3.1.2. Magnetic Characterization

**Figure 3:**
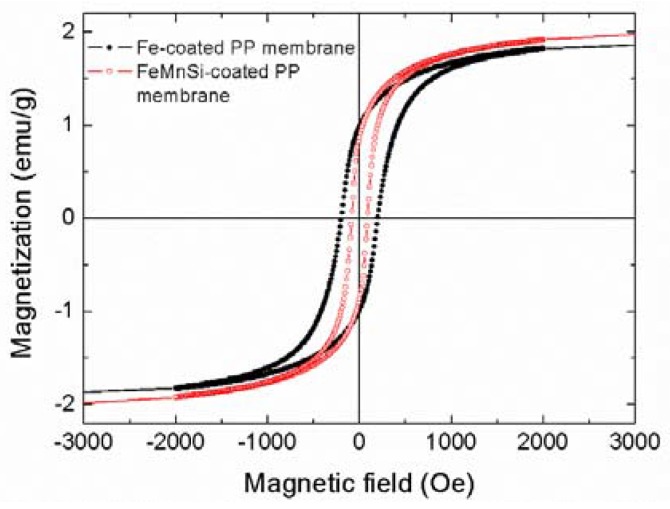
Room-temperature hysteresis loops of Fe- and FeMnSi-coated PP membranes.

The hysteresis loops of the PP membranes coated with Fe and FeMnSi (Figure 3) exhibit similar magnetization saturation (*M_S_*) values (~2 emu g^−1^). Note that the overall mass of the hybrid material (membrane plus coating) was taken into account for normalization; thus, resulting in a much smaller *Ms* value than expected (*M_s_*_,Fe_ = 217 emu g^−1^). However, the coercivity (*H_c_*) of the Fe-14Mn-4Si alloy (~90 Oe) is lower than that of pure Fe (~200 Oe). According to these values, they can be classified as soft-magnetic materials. The soft magnetic behavior of these coatings (in particular, their relatively high magnetic susceptibility), ensures that they might eventually be manipulated (magnetically-guided) using moderate external magnetic fields (easy to be applied using conventional electromagnets, for example).

#### 3.1.3. Biodegradability

The nature and quantity of metallic ions released from the Fe- and FeMnSi-coated membranes after incubation in HBSS solution for 15 days at 37 °C (Table 2) were evaluated by ICP analysis. For comparison purposes, a PP membrane (without coating) was also immersed in HBSS solution. The concentration of Fe ions released from the Fe-coated PP membrane and from the pristine PP membrane was always below the detection limit of the technique. Meanwhile, both Mn and Fe (in much larger amount) were detected in the HBSS solution in contact with the FeMnSi-coated PP membranes. These results indicated that the FeMnSi nanocoating degraded faster than pure Fe, probably as a result of the Mn addition. Figure S2 displays SEM images of the Fe- and FeMnSi- coated PP membranes after immersion in HBSS for 15 days. The morphology of the Fe-coated PP membrane remained almost the same as before the immersion (Figure 1), keeping the porous structure unaltered. On the contrary, the morphology of the FeMnSi-coated PP membranes changed after incubation, showing a spongy appearance.

#### 3.1.4. Cytotoxicity Results

**Figure 4:**
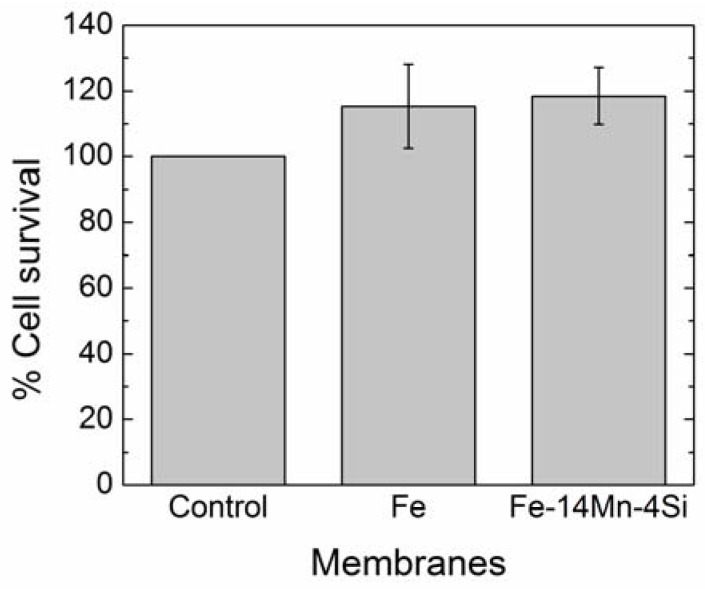
Cell viability measured with MTT cell proliferation assay protocol for the control, Fe- and FeMnSi-coated membranes.

To assess cell viability after one week of culture in the presence of the Fe- and FeMnSi-coated PP membranes, the MTT cytotoxicity assay was carried out. Since the values obtained were slightly higher for cell cultures in presence of Fe- and FeMnSi-coated PP membranes than for control cells, it can be concluded that neither Fe nor FeMnSi nanocoatings are cytotoxic (Figure 4). This increase could be attributed to Fe released during the experiment, which could increase the metabolic activity [26].

### 3.2. Transferrin-Alexa Fluor 488 Functionalization of the FeMnSi-Coated Membranes

**Figure 5:**
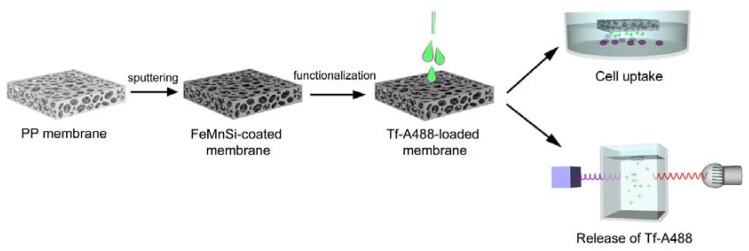
Schematic picture of the functionalization of the PP membranes process.

Taking into account the previous results (morphology, structural properties, biodegradability, and cell viability results), transferrin-Alexa Fluor 488 functionalization studies were carried out on the FeMnSi-coated membrane as this composition showed enhanced biodegradability and was proven not to be toxic. A schematic picture of the whole process is shown in Figure 5.

**Figure 6:**
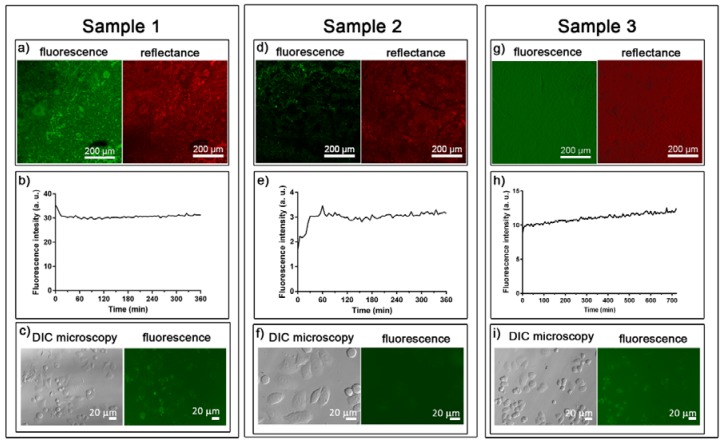
Fluorescence and reflectance images of (**a**) Sample 1; (**d**) Sample 2; and (**g**) Sample 3. Transferrin kinetic cumulative release of (**b**) Sample 1; (**e**) Sample 2 and (**h**) Sample 3. SKBR-3 cells observed under differential interference contrast (DIC) microscopy and fluorescence microscopy at 24 h of incubation for (**c**) Sample 1; (**f**) Sample 2, and (**i**) Sample 3.

Information about the distribution of the Tf-AF488 inside the porous membranes was provided by CSLM. Fluorescence and reflectance mode confocal images of Sample 1 (Figure 6a) point toward an inhomogeneous distribution of the Tf-AF488 over the surface. Tf-AF488 cumulative release kinetics (Figure 6b) showed that the Tf-AF488 was immediately released after placing the loaded membrane in the media (when the highest fluorescence intensity was observed). After the peak, fluorescence decayed during the next 15 min until it stabilized. It is worth mentioning that when the membrane was placed inside the fluorimeter cuvette, it remained on top of the media. As the fluorescence reader was located just below, a high concentration of Tf-AF488 was detected in this zone immediately after placing the membrane, as a result of the extremely fast release. Afterwards, the concentration of TF-AF488 began to balance along the cuvette until it reached an equilibrium. This can explain the fluorescence decay observed at the beginning of the test. Accordingly, uptake tests demonstrated that green fluorescence, belonging to Tf-AF488, was present inside of the cells only after 4 h of incubation (Figure S3d). After 24 h of incubation (Figure 6c and Figure S3f), fluorescence had increased and nearly all the cells showed a green spotted fluorescence pattern that corresponds to the Tf-AF488 accumulated in the endosomes and lysosomes [27].

With the aim to slow down the kinetics of release, Sample 2 was washed with HBSS after loading to eliminate the excess of Tf-AF488 accumulated onto the surface of the membrane, and a stopper of maltodextrin and gelatin was added on top of the membrane to delay the Tf-AF488 release (Table 1). Regarding Tf-AF488 distribution, confocal images (Figure 6d) indicated also a non-homogeneous distribution of Tf-AF488. However, the cumulative release kinetics (Figure 6e) showed sustained release during the first hour. Nonetheless, the cargo yield was low, as corroborated with the uptake studies in which only a diffuse fluorescence could be appreciated after 24 h of incubation (Figure 6f).

**Figure 7:**
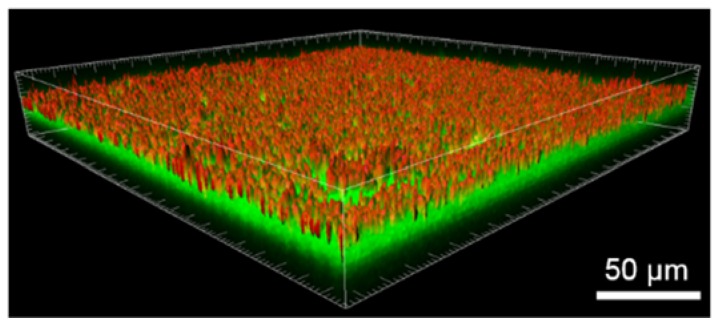
3D reconstruction obtained from CSLM images showing the distribution of Tf-AF488, in green, and the structural image of the PP membrane, in red (Sample 3).

To increase the cargo yield while keeping the progressive release achieved in Sample 2, Sample 3 was consecutively loaded three times to allow more Tf-AF488 to penetrate inside the membrane. Confocal imaging in fluorescence mode revealed that Tf-AF488 homogeneously covered the entire surface of the platform (Figure 6g). 3D image reconstruction, together with confocal microscopy, offer great advantages for studying complex nanocomposites by providing valuable 3D information. The isosurface module of Imaris 3D photoluminescence (3D-PL, green) and the structural (reflection mode, red) image of the porous Fe-14Mn-4Si membrane (Figure 7) showed a homogeneously distributed Tf-AF488 within the membrane. Regarding cellular uptake, no fluorescence could be detected at 4 h of incubation but, at 24 h of incubation (Figure 6i), Tf-AF488 fluorescence could be observed in many cells, similarly to the results observed in Sample 1 after 4 h of incubation.

Among the two replicates produced for each approach, differences could be observed for the cargo yield, especially for Samples 2 and 3, but all the replicates exhibited the same release pattern, evidencing the reproducibility of the loading conditions and Tf-AF488 release. Moreover, fluorescence was barely detectable on the membranes at the end of the fluorometric test, confirming the complete release of Tf-AF488 (Figure S4).

For fixed bio-applications, the presence of the FeMnSi covering the PP membrane might seem unclear. However, it has been shown that the release of certain molecules can be modulated by the action of an external magnetic stimuli. Concerning the use of these materials in micro-robotic applications, the issue of the size needs to be considered. Given that erythrocytes take on average 60 s to complete one cycle of circulation [28], the FeMnSi-coated PP membranes, once introduced into the circulatory system, could reach the target tissue before all of the cargo is released. Once there, they could be retained by an externally applied magnetic field, enhancing the specific accumulation of the cargo in the target tissue. In order to be able to circulate through the blood vessels, the membranes should be, in any case, miniaturized to a micrometric scale. We are currently developing a top-down approach to obtain micrometric FeMnSi-coated PP membranes able to circulate through arteries and veins.

## 4. Conclusions

The results presented in this study demonstrate that macroporous FeMnSi-coated PP membranes are interesting organic-inorganic materials for biomedical purposes. By using a physical deposition method (sputtering), macroporous PP membranes are coated with a ternary alloy. Of note, both components, the FeMnSi alloy nanocoating and the PP polymer, are biodegradable. Indeed, FeMnSi shows higher degradability in HBSS than pure Fe and is not cytotoxic. The macroporous FeMnSi-coated PP membranes have been functionalized with Tf-AF488 molecule using various protocols, which provide a different balance between cargo yield and release. Thus, their functionalization can be adapted to host molecules that require different concentration or release kinetics to obtain an optimal response. Interestingly, after the cargo is released, both the FeMnSi alloy and the polymer would degrade within a reasonable timescale without the need of a post-extraction surgery.

## Figures and Tables

**Table 1:** Summary of the different procedures (in chronological order) followed to incorporate Tf-AF488 in the FeMnSi-coated PP membranes for three different cases (denoted as Samples 1, 2 and 3).

Steps (# of Times)	Sample 1	Sample 2	Sample 3
Loading with Tf-AF488	1	1	3
Cleaning with HBSS	0	1	1
Drying under vacuum	1	1	1
Stopper	0	1	2
Drying under vacuum	0	1	1

**Table 2:** Concentration of Fe and Mn released from the Fe- and FeMnSi-coated PP membranes after immersion in HBSS solution for 15 days at 37 °C.

Membrane Type	[Fe] (μg L^−1^)	[Mn] (μg L^−1^)
PP membrane	<10	<10
Fe	<10	<10
Fe-14Mn-4Si	19 ± 6	17 ± 2

## Supplementary Materials

The following are available online at www.mdpi.com/2079-4991/7/7/155/s1, Figure S1: X-ray diffraction patterns of Fe (black line) and Fe-14Mn-4Si (red line) sputtered on flat Si/Ti/Cu substrates. The first diffraction peak (*) belongs to the Cu substrate and the second one (+) to Fe (110) BCC Im$\bar{3}$m, Figure S2: SEM images of (a) Fe- and (b) FeMnSi-coated PP membranes after incubation in HBSS for 15 days, Figure S3: SKBR-3 cells observed under differential interference contrast (DIC) microscopy and fluorescence microscopy after different times of incubation in the presence of Sample 1: (a,b) control cells, (c,d) cells incubated for 4 h, (e,f) cells incubated for 24 h. Scale Bar, 20 µm, Figure S4: Fluorescence image of Sample 3 after the fluorimetric assay indicating total absence of Tf-AF488.
