# Supplementary material for: Biodegradable FeMnSi Sputter-Coated Macroporous Polypropylene Membranes for the Sustained Release of Drugs
Source: Nanomaterials (Basel). 2017 Jun 24;7(7):155. doi: 10.3390/nano7070155 (PMC5535221; doi:10.3390/nano7070155)
Supplement: Supplementary file 1 [file nanomaterials-07-00155-s001.pdf]

Supplementary Nanomaterials: Biodegradable FeMnSi Sputter-Coated Macroporous Polypropylene Membranes for the Sustained Release of Drugs

Jordina Fornell [1], Jorge Soriano [2], Miguel Guerrero [1,*], Juan de Dios Sirvent [1], Marta Ferran-Marqués [1], Elena Ibáñez [2], Leonardo Barrios [2], Maria Dolors Baró [1], Santiago Suriñach [1], Carme Nogués [2,*], Jordi Sort [1,3,*] and Eva Pellicer [1]

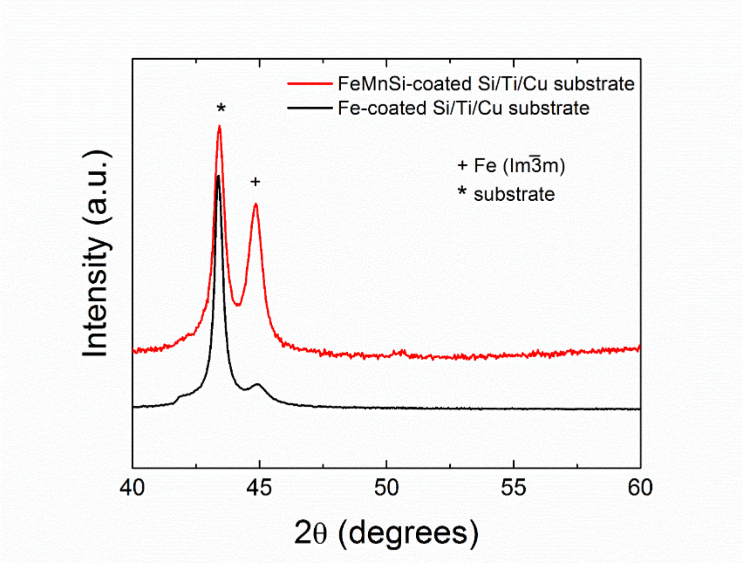

Figure S1. X-ray diffraction patterns of Fe (black line) and Fe-14Mn-4Si (red line) sputtered on flat Si/Ti/Cu substrates. The first diffraction peak (*) belongs to the Cu substrate and the second one (+) to Fe (110) BCC Im$\bar{3}$m.

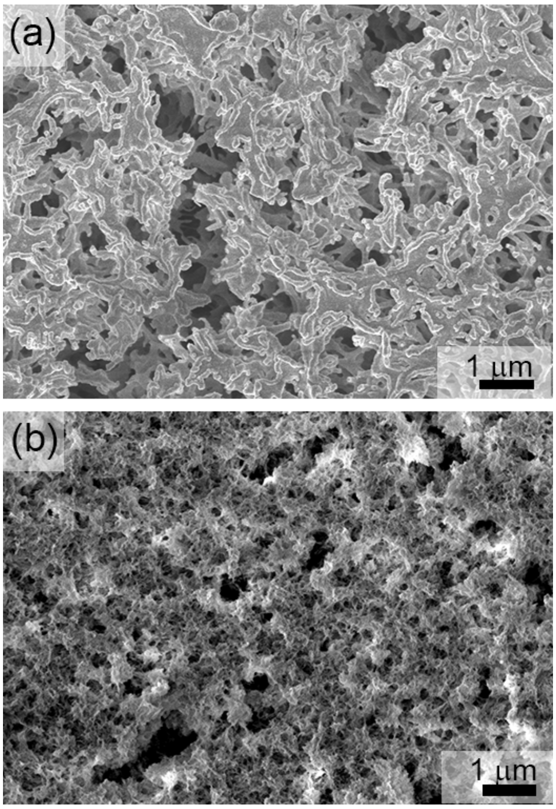

Figure S2. SEM images of (**a**) Fe- and (**b**) FeMnSi-coated PP membranes after incubation in HBSS for 15 days.

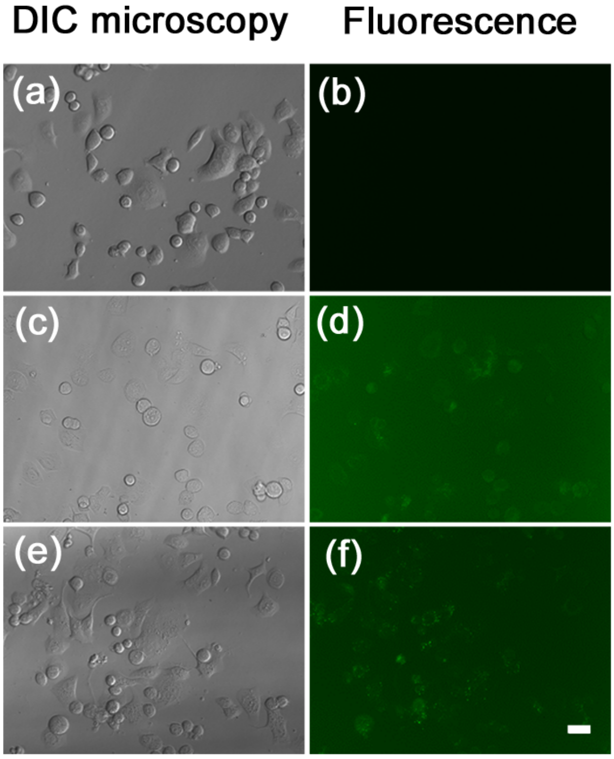

Figure S3. SKBR-3 cells observed under Differential Interference Contrast (DIC) microscopy and fluorescence microscopy after different times of incubation in the presence of Sample 1: (**a**,**b**) control cells, (**c**,**d**) cells incubated for 4 h, (**e**,**f**) cells incubated for 24 h. Scale Bar, 20 µm.

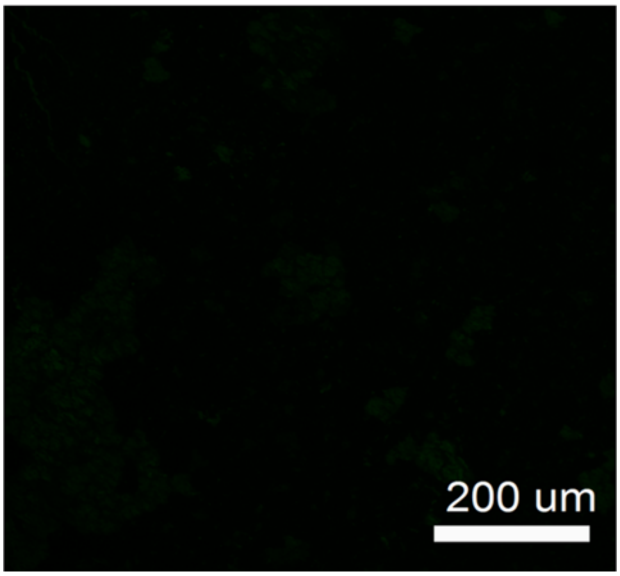

Figure S4. Fluorescence image of Sample 3 after the fluorimetric assay indicating total absence of Tf-A488.
